# Decadal changes in biomass and distribution of key fisheries species on Newfoundland’s Grand Banks

**DOI:** 10.1371/journal.pone.0300311

**Published:** 2024-04-01

**Authors:** Raquel Ruiz-Diaz, Maria Grazia Pennino, Jonathan A. D. Fisher, Tyler D. Eddy

**Affiliations:** 1 Centre for Fisheries Ecosystems Research, Fisheries & Marine Institute, Memorial University, St. John’s, Newfoundland and Labrador, Canada; 2 Spanish Institute of Oceanography (IEO, CSIC), Madrid Oceanographic Center, Madrid, Spain; Swedish University of Agricultural Sciences and Swedish Institute for the Marine Environment, University of Gothenburg, SWEDEN

## Abstract

Canadian fisheries management has embraced the precautionary approach and the incorporation of ecosystem information into decision-making processes. Accurate estimation of fish stock biomass is crucial for ensuring sustainable exploitation of marine resources. Spatio-temporal models can provide improved indices of biomass as they capture spatial and temporal correlations in data and can account for environmental factors influencing biomass distributions. In this study, we developed a spatio-temporal generalized additive model (st-GAM) to investigate the relationships between bottom temperature, depth, and the biomass of three key fished species on The Grand Banks: snow crab (*Chionoecetes opilio*), yellowtail flounder (*Limanda ferruginea*), and Atlantic cod (*Gadus morhua*). Our findings revealed changes in the centre of gravity of Atlantic cod that could be related to a northern shift of the species within the Grand Banks or to a faster recovery of the 2J3KL stock. Atlantic cod also displayed hyperaggregation behaviour with the species showing a continuous distribution over the Grand Banks when biomass is high. These findings suggest a joint stock assessment between the 2J3KL and 3NO stocks would be advisable. However, barriers may need to be addressed to achieve collaboration between the two distinct regulatory bodies (i.e., DFO and NAFO) in charge of managing the stocks. Snow crab and yellowtail flounder centres of gravity have remained relatively constant over time. We also estimated novel indices of biomass, informed by environmental factors. Our study represents a step towards ecosystem-based fisheries management for the highly dynamic Grand Banks.

## Introduction

Stock assessments aim to evaluate the status of a population by evaluating biomass and fishing mortality relative to reference points to define catch limits [1]. Time series of biomass indices are often used to calibrate stock assessment models, primarily derived from fisheries-independent data collected from scientific surveys. These surveys commonly employ a stratified-random sampling design to generate estimates of absolute biomass by using area-swept information [2, 3]. This involves dividing the study area into different strata based on specific characteristics, such as depth or habitat type. Within each stratum, random samples are collected. Random stratified sampling increases the precision of the estimates when the population is homogenously distributed among strata. However, variability in habitat preference may exist within strata, compromising the robustness of this approach [4]. This approach also requires all strata to be sampled at each sampling event, which is not always possible due to inclement weather, broken ships, among others.

Spatial correlation is a common feature of fisheries data. It occurs when observations collected at different locations are not independent of each other, as nearby things tend to be more connected than distant things [5]. If unaccounted for, spatial correlation can lead to biased estimates of biomass and abundance [6]. This also applies to the correlation of objects through time [7]. Spatio-temporal generalized mixed effect models (GLMMs) and generalized additive models (GAMs) can account for spatial heterogeneity found in fisheries survey data [8]. The latter are more flexible because they are fit using smoothing spline terms, making them especially useful for addressing non-linear relationships [9]. These models explicitly account for both spatial and temporal correlations in a dataset [10], and can incorporate information about environmental variables that may be driving species biomass and distribution [11]. Another advantage of these methods is that they employ spatial interpolation throughout the region of interest, overcoming incomplete sampling issues [9]. Spatio-temporal models can improve predictions for areas and years with little or no data and can be more precise than with design-based methods (i.e., strata-based index) and conventional GLMMs and GAMs (i.e., without spatio-temporal effects) [12, 13].

Our study focuses on the practical application of spatio-temporal Generalized Additive Models (st-GAMs) [14] to understand the biomass dynamics and distributional changes of snow crab (*Chionoecetes opilio*), yellowtail flounder (*Limanda ferruginea*), and Atlantic cod (*Gadus morhua*) on The Grand Banks of Newfoundland. The Grand Banks is a highly productive region where two distinct water masses, the Labrador Current and the Gulf Stream, converge. The Labrador Current brings fresh and nutrient-rich waters, while the Gulf Stream carries warmer and saltier waters. This combination of conditions makes The Grand Banks a dynamic and highly variable ecosystem [15, 16]. The Grand Banks experienced a regime shift in the early 1990s characterized by the collapse of the Atlantic cod, yellowtail flounder and other groundfish species. However, species such as snow crab and northern shrimp proliferated during that time [17]. The shift was attributed to a combination of factors, including overfishing and changes in environmental conditions [18]. In recent years, the species in this study exhibited different dynamics, with snow crab experiencing a significant decline, Atlantic cod remaining persistently low and yellowtail flounder recovered to pre-collapse levels. Currently, the yellowtail flounder fishery is operating as a Marine Stewardship Council (MSC) certified fishery [19].

The Grand Banks constitute an independent ecosystem production unit within the Newfoundland and Labrador Shelf, characterized by high ecosystem productivity and a well-defined marine community [17, 20]. We used this ecosystem production unit as our spatial scale of focus and examined how it relates to the management units of the study species. Our study contributes to the evolving landscape of ecosystem-based fisheries management through the application of advanced modeling techniques to uncover spatio-temporal patterns in snow crab, yellowtail flounder and Atlantic cod dynamics. These species were selected based on their variable responses post cod collapse, their cultural and economic fisheries importance and their distinct movement behaviors. Gadoids are streamlined swimmers, flatfish are bottom-dwellers that perform undulatory movements and snow crab have legs that allow for lateral movement. These adaptations are shaped by the specific ecological niches and lifestyles of each species. By incorporating environmental variables into our analysis (bottom temperature and depth) and considering spatial heterogeneity, we explored species-habitat associations and calculated environmental-informed biomass indices that can be used in stock assessment models. Finally, we assessed the effect of fishing on the environmental-informed biomass indices.

## Material and methods

### Data sources

#### Multispecies survey

**Fig 1:**
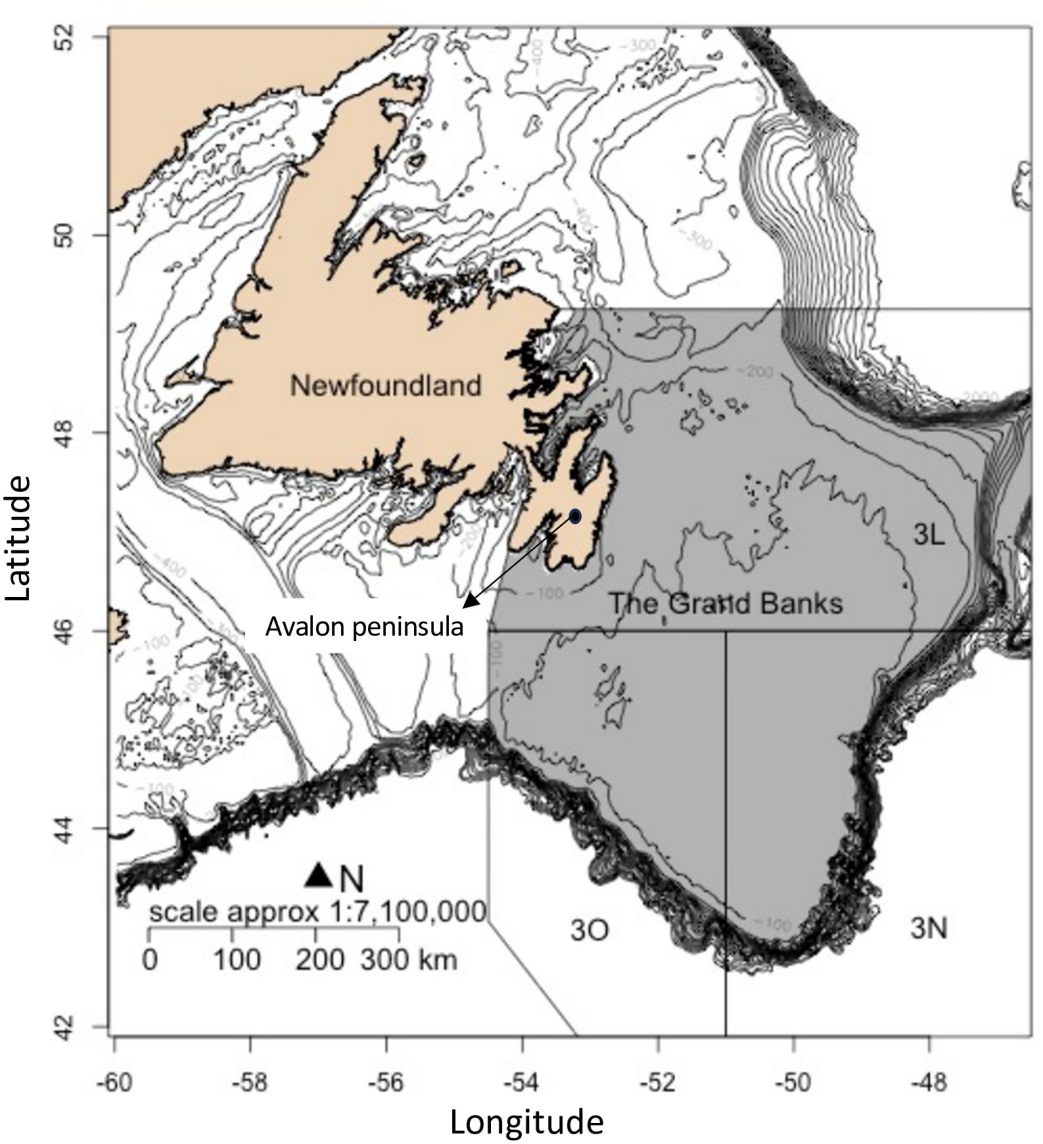
The Grand Banks of Newfoundland (grey). Solid lines indicate the management unit boundaries of the North Atlantic Fisheries Organization (NAFO) divisions (3L, 3N and 3O).

Fisheries and Oceans Canada (DFO) have been conducting annual stratified-random multispecies trawl surveys on The Grand Banks, located in the Northwest Atlantic since 1971 [21] (Fig 1). The survey is conducted in spring and fall and has changed sampling gear and coverage over time. Fall surveys, particularly in 2004–06, have issues with an absence of deep sets, reduced coverage and timing extensions due to vessel breakdowns and unplanned changes [22]. In contrast, the spring survey (April-June) has had more consistent coverage [mean observations of 282.5 per year], although the 2006 and 2017 surveys had lower coverage [194 and 191 observations, respectively]. To limit variability introduced by changes in the surveys, we utilized data from surveys conducted in spring (6,780 observations in total). Since 1996, spring surveys have been conducted with the *CCGS Alfred Needler* vessel and *Campelen 1800 shrimp trawl* gear, sampling up to 732 meters depth. The trawl survey collects data on the abundance, size, and biomass of numerous groundfish and shellfish species, in addition to other biological information (i.e., size, maturation status, body condition, stomach contents) [21].

Biomass data were used to create two response variables for each species: presence/absence and conditional-to-presence-biomass. Abiotic explanatory variables were bathymetry (here called depth) and bottom temperature, also obtained from the DFO bottom trawl survey (Table 1). These covariates were selected based on their well-known relationship with distribution and productivity of the study species [23–25]. The relationship between the study species and salinity is less understood in this region and was not considered here. Finally, we assumed that expected fish biomass is proportional to the area surveyed (referred to as swept area); thus we included swept area (log-transformed) as offset in all the models to account for the effort.

**Table 1:** List of response and explanatory variables included in the st-GAM.

	Name	Description	Units
Explanatory variable	Depth	Bathymetry at sampling location	metres
	Temperature	Bottom Temperature at sampling location	ºC
Response variable	Occurrence & conditional-to-presence biomass	Presence/absence and biomass of snow crab (*Chionoecetes opilio*)	Kg.tow-1 (live weight)
	Occurrence & conditional-to-presence biomass	Presence/absence and biomass of Atlantic cod (*Gadus morhua*)	Kg.tow-1 (live weight)
	Occurrence & conditional-to-presence biomass	Presence/absence and biomass of yellowtail flounder (*Limanda ferruginea*)	Kg.tow-1 (live weight)

#### Prediction grid

**Fig 2:**
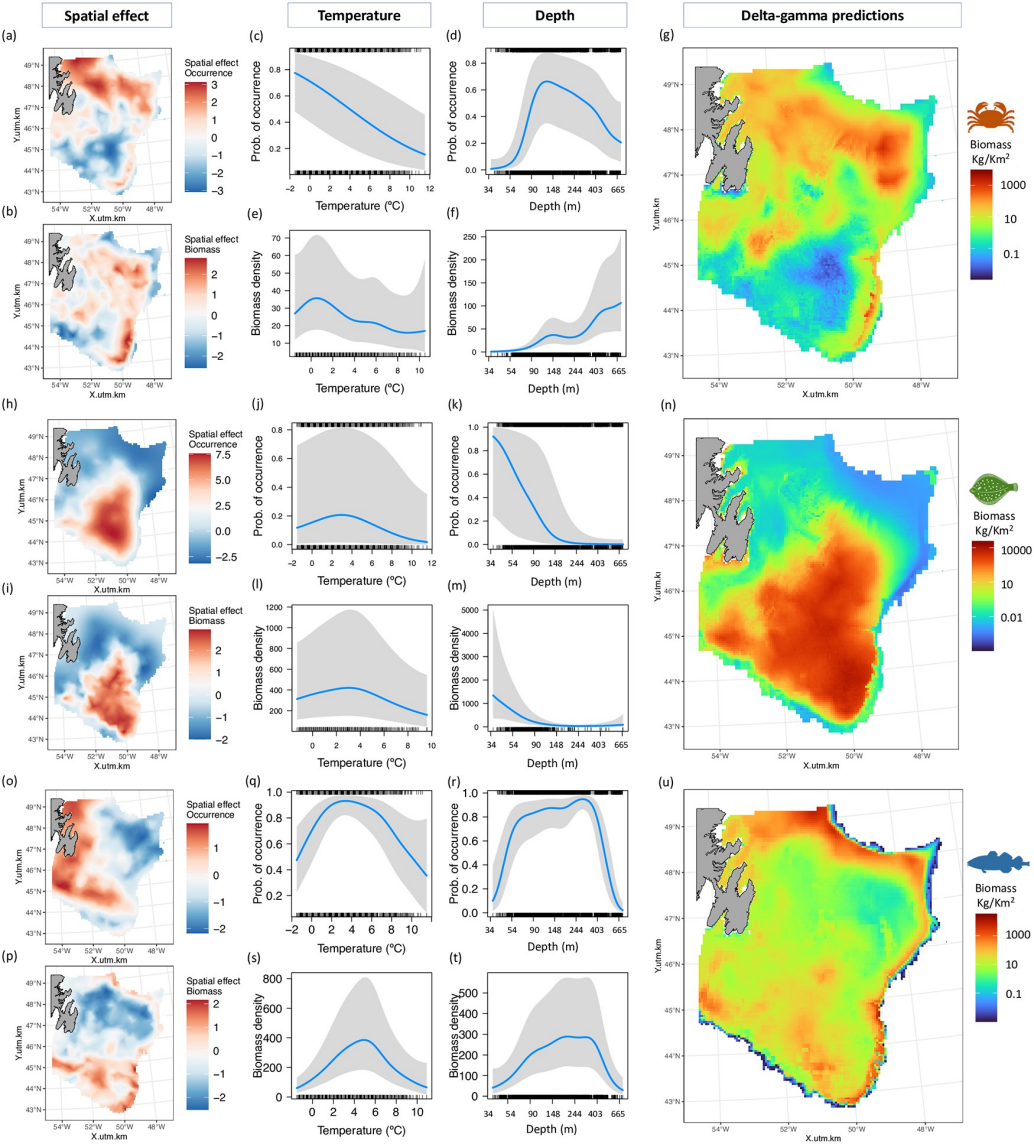
Spatial random field deviations of snow crab probability of occurrence (a) and biomass (b), yellowtail flounder probability of occurrence (h) and biomass (i), and Atlantic cod probability of occurrence (o) and biomass (p). Temperature smoothed effects on snow crab probability of occurrence (c) and biomass (d), yellowtail flounder probability of occurrence (j) and biomass (k), and Atlantic cod probability of occurrence (q) and biomass (r). Depth smoothed effect on snow crab probability of occurrence (e) and biomass (f), yellowtail flounder probability of occurrence (l) and biomass (m), and Atlantic cod probability of occurrence (s) and biomass (t). Note that depth has been log transformed. Biomass density units are kg/25 km^2^. Delta gamma combined biomass prediction of snow crab (g), yellowtail flounder (n) and Atlantic cod (u) over the Grand Banks. Predictions made on a 5x5 km grid.

We created a 5x5 km grid for The Grand Banks with 274,461 grid points. We used gridded bathymetry data with spatial resolution of 15 arc seconds (≈0.004 º) obtained from the General Bathymetric Chart of the Oceans (GEBCO) project (https://www.gebco.net) and manipulated it with the R package *marmap* [26] to extract depth values at each grid point. Rasters of bottom temperature data were provided by DFO. These rasters were created using data from different sources (DFO multispecies survey, Atlantic Zone Monitoring Program (AZMP) hydrographic campaigns, International Oceanographic Campaigns (IOC), ARGO program, etc) [27]. All data were vertically averaged in 5 m bins, and a linear interpolation was applied to fill missing bins. We selected the data corresponding to the spring season (April-June) for which data were averaged on a regular 0.1° x 0.1° grid. Horizontal linear interpolation was applied to overcome missing data on grid cells (full description in [27]). We tested the correlation between these databases (i.e., GEBCO and DFO temperature interpolation) with trawl station observations to assess accuracy (Figs 1 and 2 S1 Appendix).

#### Fishing data

Finally, fishing data aggregated at division level were obtained from the NAFO STLATLANT database. Strata-based indices of snow crab [28], yellowtail flounder [29] and Atlantic cod [30] were collected and compared to the new environmental-informed indices.

### Spatio-temporal modelling

An exploratory analysis highlighted that species biomass data have two main features, namely strong spatial and temporal dependence and a large proportion of observed zeros (i.e., zero inflated data). To address this, we developed a delta gamma generalized additive model (GAM) using the R-package *sdmTMB* [31]. This separately analyzes species occurrence (biomass information is transformed to 0 and 1 according to species absence and presence, respectively) and conditional-to-presence biomass (observations with positive biomass values) and combines both predictions in a final estimate of biomass [8]. Delta gamma models are commonly found in the literature to perform analysis similar to this one [32, 33]. We considered *Z*(*s*,*t*) to be the spatiotemporally distributed occurrence and *Y*(*s*,*t*) the conditional-to-presence biomass at location *s* and time *t*. The final model formulation is described as follows (Formula 1):

$$Z(s,t)\sim \;Bernoulli\left(\pi \left(s,\;t\right)\right)$$ (1)

Y(s,t)~Gamma(μ(s,t),ϕ)

$$logit(\pi \;\left(s,\;t\right))=\beta_{z}+\;Y_{i}+\sum_{i=1}^{I}\;{f_{i\;}(X}_{i\;}(s,t))+\;{\text{V}}_{z}\;\left(s,t\right)$$

$$\text{log}(\mu \left(s,t\right))=\beta_{Y}+\;Y_{i}+\sum_{i=1}^{I}{f_{i\;}(X}_{i\;}(s,t))+\;{\text{V}}_{Y}\;\left(s,t\right)$$

where *π* (*s*, *t*) represents the probability of occurrence at location *s* and time *t*; and *μ*(*s*,*t*) and *ϕ* are the mean and variance of the conditional-to-presence biomass, respectively. The linear predictors, which represent the intercept of each variable associated to the parameter *π* (*s*, *t*) and *μ* (*s*, *t*), are represented by *β*_*z*_ and *β*_*Y*_, respectively. Survey year was added as a fixed effect in our model (*Y*_*i*_). *f*() represents any function applied to the covariates (*X*_*i*_), which in the present study were smoothing terms (p-splines). *V*_*z*_(*s*, *t*) and *V*_*y*_(*s*, *t*) refer to the spatio-temporal structure of the occurrence and conditional-to-biomass model, respectively.

**Fig 3:**
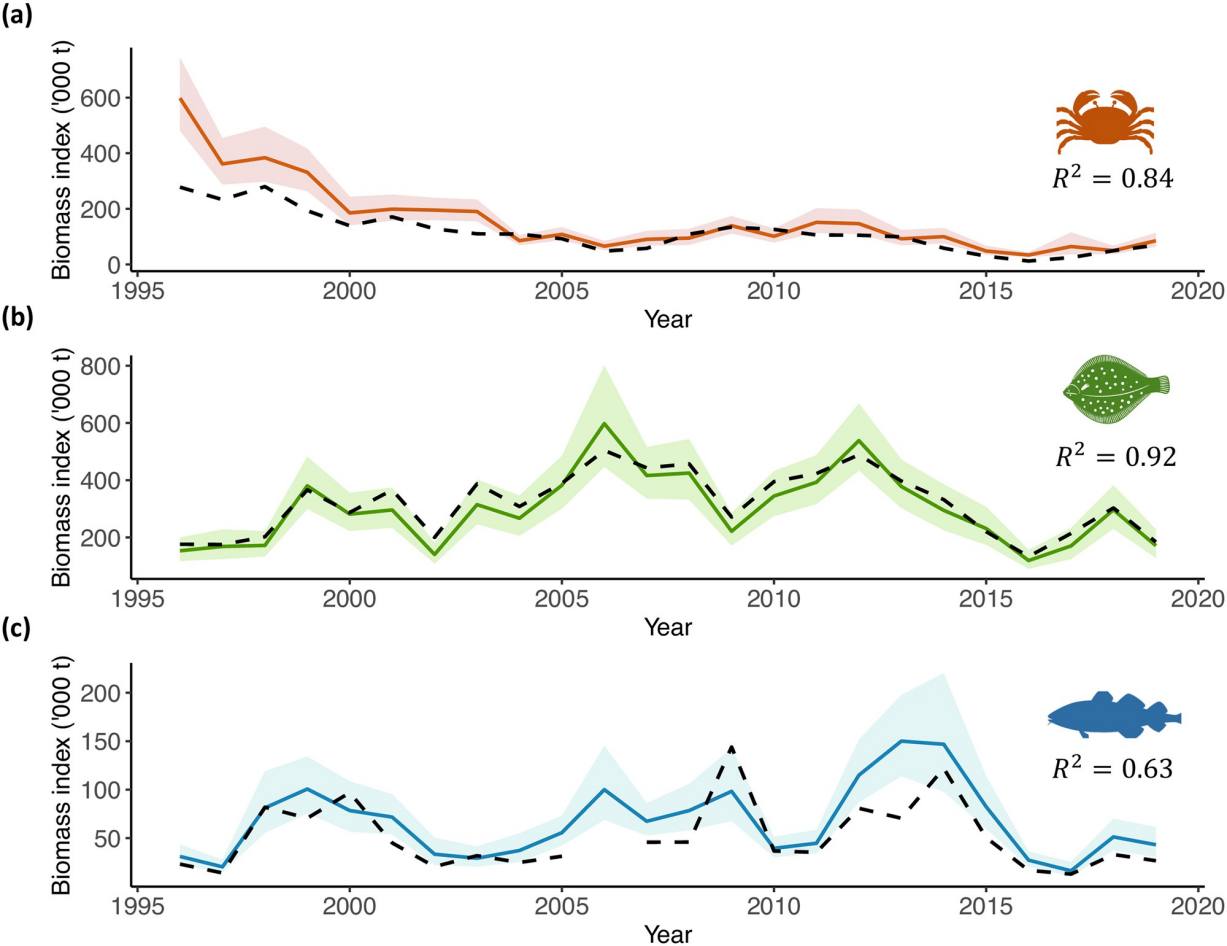
Biomass indices of snow crab (orange), yellowtail flounder (green) and Atlantic cod (blue) on The Grand Banks of Newfoundland estimated from the spatio-temporal delta gamma GAM. Shaded areas indicate the 95% confidence interval. Black dashed line indicates the strata-based index of the species (units in tonnes x1000). *R*^2^ is the coefficient of determination.

*sdmTMB* relies on the integrated nested Laplace approximation (INLA) to discretized the space by defining a Delaunay triangulation mesh, which in turn creates an artificial set of neighbors over the study area, and Gaussian Markov random fields to model spatial dependencies between observations [31, 34]. Our approach involved constructing a Delaunay triangulation mesh with a defined minimum distance of 20 km, resulting in a mesh comprising 467 vertices (Fig 3 in S1 Appendix). This choice was carefully considered, accounting for the spatial distribution of sampling locations and aiming to strike a balance between computational efficiency and predictive accuracy. To test which model performs better, we evaluated the spatial effect by running a model with and without the spatial component. The spatio-temporal component was included as: 1) a first-order autoregressive effect (AR1), which has a parameter (*rho)* that regulates the degree of autocorrelation between random field deviations from one year to the next; 2) a random walk (RW) and 3; an independent and identically distributed (iid), in which the random fields are assumed to be independent across time steps, to test which one performs better.

### Model selection and validation

We calculated Pearson’s rank correlation index and the variance inflation factor (VIF) of the covariates before model runs. This helped avoid correlation and collinearity among explanatory variables [35]. We did not find any substantial correlation among the covariates (*R* < 0.6 and *VIF* < 3), allowing us to proceed with including depth and bottom temperature in the st-GAM. To model the non-linear relationship between explanatory and response factors, covariates were included to the model as random factor with a smoothing term (p-splines) [31]. Depth was log-transformed for better model convergence.

To assess the importance of bottom temperature, depth, and the spatial component on species spatial distribution, Akaike weights were used instead of stepwise variable selection, as they account for model selection uncertainty [36, 37]. By summing these weights, the relative importance of each variable can be estimated. To test model predictability, we carried out a k-fold cross-validation in which data were randomly split in k = 4 folds of equal size. In each iteration, one of the folds is held to test the data, while the other 3 are used to train the model. Then, we used the expected log pointwise predictive density (ELPD) to evaluate model predictive accuracy [31, 38].

To validate models, residuals were evaluated to ensure that spatial patterns were not detected and that residuals were normally distributed (S2 Appendix).

### Biomass index and centre of gravity calculation

Once models were fit, we predicted species biomass over the study area using a 5x5 km grid. We summed up the densities calculated in the predictions and multiplied them by cell area to calculate the biomass index. In the case of snow crab, species catchability in the bottom trawl survey was found to be lower than 1 [39]. To account for this issue, a conversion factor was calculated using a Delury depletion regression analysis on fishery catch rate data from logbooks from 2000 to 2016 [40] and biomass estimates were adjusted by a factor of 0.126. Agreements between the new biomass indices (spatially aggregated biomass data) and strata-based indices (strata aggregated biomass data) reported in the stock assessment of snow crab [28], yellowtail flounder [29] and Atlantic cod [30] were assessed by using the coefficient of determination, R^2^. For snow crab, the strata-based indices were estimated using fall survey data, whereas our environmental-informed indices were estimated using spring data. The models used in the assessment of all stocks except 3NO Atlantic cod require biomass indices as input (snow crab assessment uses biomass trends, yellowtail flounder a Schaefer surplus production model and 2J3KL Atlantic cod a state-space model). Therefore, the new biomass indices developed in the present study are comparable and could be used in the assessment of the species.

Finally, we estimated the centre of gravity of the populations using the following (Formula 2):

$$CG_{year}=\frac{\sum_{i=1}^{n}x_{i}w_{i}}{\sum_{i=1}^{n}w_{i}}$$ (2)

where *x*_*i*_ is the location (x or y-coordinates) of the grid cell, *w*_*i*_ is the species biomass estimated at each grid cell *i*, and *n* is the total number of grid points in the study area (*n* = 274,461) [41]. We also calculated the species centre of gravity directly from the data using mean weight.

### Fishing impact

We were unable to account for the impact of fishing due to a lack of spatially resolved fishing effort data that could be integrated into the model. Despite this limitation, we endeavored to explore the fishing effect on species biomass using a linear regression model with species biomass at the divisional level (3L, 3N, and 3O) as the response variable and fishing catch data aggregated at the same level (NAFO STLATLANT database) as the explanatory variable. We only investigated yellowtail flounder and snow crab because the commercial Atlantic cod fishery on The Grand Banks has remained closed since 1992 due to the slow recovery after its collapse, with species being captured as bycatch and in recreational (known as the food fishery) and small vessel, inshore commercial fisheries [42].

## Results

For all species, models that included spatial effects produced better fits. Similarly, models that included bottom temperature and depth effects as covariates performed better (Table 2). Our results showed that the spatial effect explained most of the variability in the biomass data, followed by depth and temperature, respectively (Table 2-*Δ*AIC values). We also tested different spatio-temporal configurations (AR1, RW and iid) and found that the autoregressive spatio-temporal structure (AR1) had higher predictive accuracy for snow crab and yellowtail flounder, while independent and identically distribution (iid) performed better for Atlantic cod (Table 2-*Δ*ELPD values). AR1 has a *rho* parameter that indicates the degree of correlation from one year to the next. We found that yellowtail flounder biomass had a *rho* = 0.73 and snow crab biomass had a *rho* = 0.71.

**Table 2:** Models used to identify the best configuration based on Akaike information criteria weights (ΔAIC) and expected log pointwise predictive density weights (ΔELPD). Model structure indicates the different configurations tested in the model. Note that bottom temperature (temp) and depth were added as smoothing terms (p-spline). df indicates the degree of freedom, AIC is the Akaike information criteria, and ΔAIC indicates differences among AICs. ELPD is the expected log pointwise predictive density, and differences among ELPDs are indicated by ΔELPD. The best model configuration is in bold.

Species	Model structure	df	AIC	ΔAIC	ELPD	ΔELPD
**Snow crab**	**biomass ~ year + temp + depth + spatial + AR**	**65**	**16165.08**	**0**	**0.066**	**0**
	biomass ~ year + temp + depth + spatial + RW	63	16227.41	-62.33	0.017	-0.048
	biomass ~ year + temp + depth + spatial + iid	63	16380.88	-215.8	0.017	-0.049
	biomass ~ year + depth + spatial + iid	59	16421.67	-256.59	-0.010	-0.077
	biomass ~ year + spatial + iid	55	16719.23	-554.15	-0.057	-0.123
	biomass ~ year + spatial	53	17429.39	-1264.31	-0.379	-0.446
	biomass ~ year + depth + temp	57	18876.37	-2711.29	-0.731	-0.797
	biomass ~ year	49	22066.03	-5900.95	-1.123	-1.190
**yellowtail flounder**	**biomass ~ year + temp + depth + spatial + AR**	**65**	**25270.75**	**0**	**-2.601**	**0**
	biomass ~ year + temp + depth + spatial + RW	63	25320.52	-49.77	-2.632	-0.031
	biomass ~ year + temp + depth + spatial + iid	63	25406.76	-136.01	-2.614	-0.013
	biomass ~ year + depth + spatial + iid	59	25416.07	-145.32	-2.646	-0.044
	biomass ~ year + spatial + iid	55	25624.18	-353.43	-2.652	-0.050
	biomass ~ year + spatial	53	25998.47	-727.72	-2.882	-0.281
	biomass ~ year + depth + temp	57	27189.61	-1918.86	-3.389	-0.788
	biomass ~ year	49	33741.03	-8470.28	-4.392	-1.791
**Atlantic cod**	biomass ~ year + temp + depth + spatial + AR	65	26026.72	0	-1.121	-0.007
	biomass ~ year + temp + depth + spatial + RW	63	26241.75	-215.03	-1.220	-0.106
	**biomass ~ year + temp + depth + spatial + iid**	**63**	**26126.11**	**-99.39**	**-1.113**	**0**
	biomass ~ year + depth + spatial + iid	59	26315.88	-289.16	-1.119	-0.006
	biomass ~ year + spatial + iid	55	26990.33	-963.61	-1.194	-0.081
	biomass ~ year + spatial	53	28139.83	-2113.11	-1.500	-0.387
	biomass ~ year + depth + temp	57	29430.96	-3404.24	-1.818	-0.697
	biomass ~ year	49	31308.14	-5281.42	-2.195	-1.082

### Spatial and covariate effects

The spatial random field represents consistent deviations in space through time that are not accounted for by depth and bottom temperature covariates. Higher spatial deviations were found in the north of The Grand Banks for snow crab occurrence, while they were higher near the nose and tail of the Banks for snow crab biomass (Fig 2a and 2b). Higher spatial deviations of yellowtail flounder probability of occurrence and biomass were both found in the southern part of The Grand Banks (Fig 2h and 2i). Higher spatial deviations of Atlantic cod occurrence were found in the western part of the Banks and in the 3O division, while spatial deviations were higher in the south (3NO division) and around The Grand Banks’ periphery for Atlantic cod biomass (Fig 2o and 2p).

Higher probability of snow crab occurrence and biomass was associated with colder temperatures, below 0 ºC (Fig 2c and 2d), and at depths of about 100 m for occurrence and 450 m for biomass (Fig 2e and 2f). For yellowtail flounder, both higher probability of occurrence and higher biomass were predicted for temperatures close to 3 ºC and shallower depths around 80 m (Fig 2j–2m). Atlantic cod probability of occurrence was predicted to be highest at temperatures close to 3 ºC and depths of 300 m (Fig 2q and 2r). Highest biomass of Atlantic cod was predicted to occur at 5 ºC and at depths between 200 and 400 m (Fig 2s and 2t).

Combined predictions of the delta gamma models indicate that snow crab biomass is higher in the north of the Banks, with a hotspot located in the northeast, and in the southeastern edge of the Banks (Fig 2g). The yellowtail flounder biomass hotspot is found south of the Banks (Fig 2n). Finally, Atlantic cod biomass is higher in the north and periphery of the Banks, as well as in the southeast (Fig 2u).

### Biomass indices

A decline in the snow crab relative biomass index was observed over time, reaching a minimum of 33.72 t in 2016 (Fig 3a). Although there has been a small recovery since then, the current biomass index of snow crab was 85% lower in 2019 than at the beginning of the time series in 1996. Yellowtail flounder relative biomass has fluctuated over time, peaking in 2006 (597.88 t), 2012 (538.31 t) and 2008 (424.83 t) (Fig 3b). However, the biomass plummeted to its lowest value in 2016 (119.96 t) and has remained relatively low since then, with a biomass of 171.12 t in 2019. Similarly, Atlantic cod biomass has fluctuated over time, reaching its highest value in 2013 (150.07 t), with smaller peaks in 1999 (100.6 t) and 2006 (100.04 t) (Fig 3c). Since 2014, the relative biomass of Atlantic cod has been on the decline, hitting a record low of 19.30 t in 2017, a similar biomass level when the stock collapsed in the mid 1990s (31.12 and 20.39 t in 1996 and 1997, respectively). As of 2019, the biomass of Atlantic cod on The Grand Banks was rather low compared to the historical series (43.17 t).

Our model effectively generated an environmental-informed biomass index that showed trends consistent with strata-based indices reported in the stock assessments of the studied species. For yellowtail flounder, our biomass index had a high correlation (*R*^2^ = 0.92), with the strata-based index falling within the confidence interval. For snow crab, the correlation with the strata-index was also high (*R*^2^ = 0.84). However, some disparities were evident between the indices, as our analysis indicates slightly higher biomass estimates, particularly at the beginning of the time series. Note that we are comparing our biomass index created using spring data with the strata-based biomass index created using fall data. Unfortunately, no established index exists for Atlantic cod in the 3LNO division since this species is considered two separate stocks (i.e., 2J3KL and 3NO). As a result, we compared our biomass index to the index used to assess the 3NO stock, as it more accurately represents cod biomass within The Grand Banks, resulting in a correlation of *R*^2^ = 0.63.

### Centre of gravity

**Fig 4:**
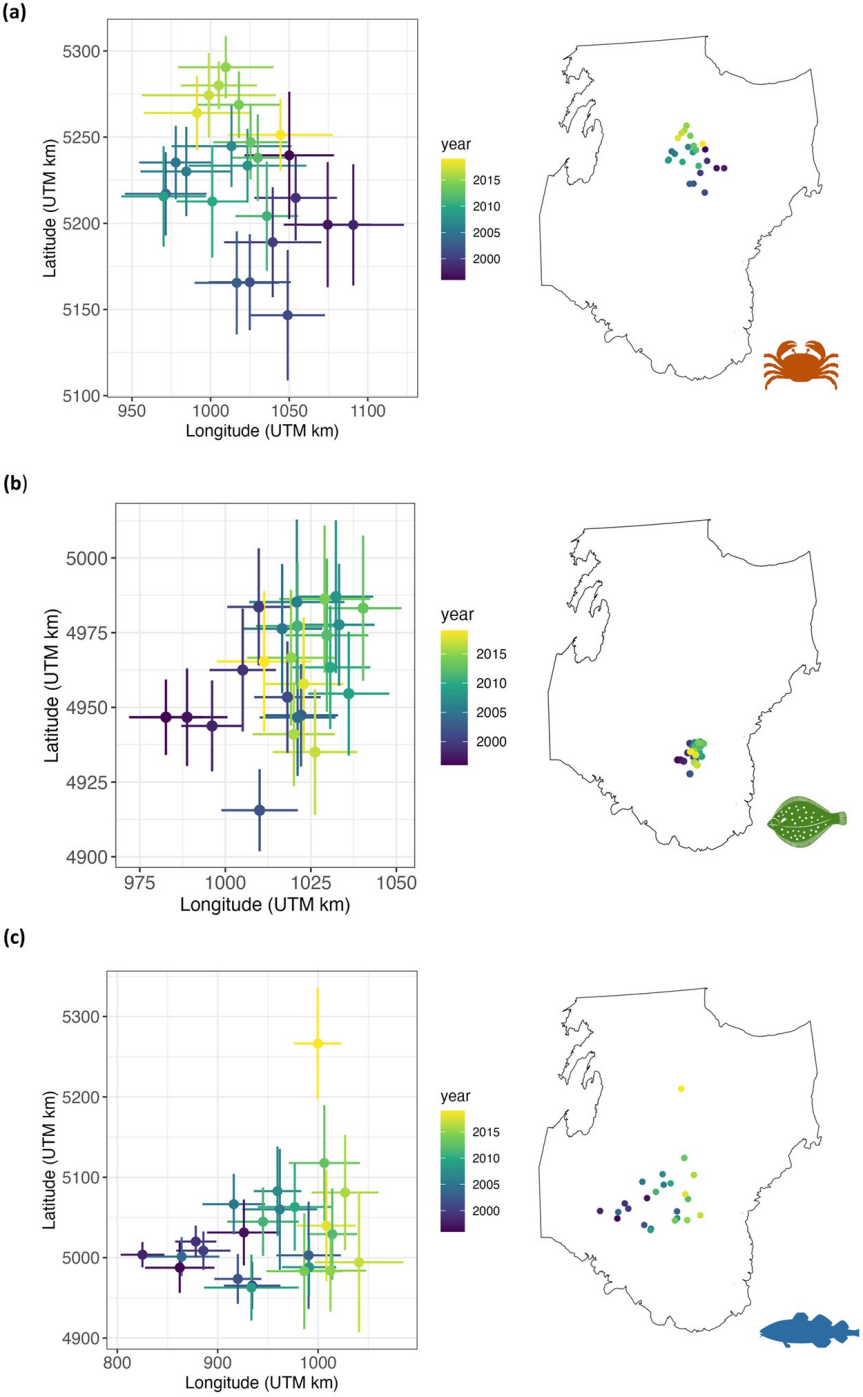
Centre of gravity of snow crab (a), yellowtail flounder (b) and Atlantic cod (c) on The Grand Banks of Newfoundland. Points indicate mean values and bars indicate variance. Colors represent years (from 1996 to 2019), with more recent years in yellow.

Centre of gravity indicates the central point of a population distribution. In the case of snow crab, the centre of gravity shifted slightly toward the northwest of The Grand Banks over time (Fig 4a). The centre of gravity of yellowtail flounder has remained relatively stable (Fig 4b). Atlantic cod had the greatest changes in centre of gravity, first moving eastward and then northward at the end of the time series (Fig 4c). We also calculated the species centre of gravity directly from the data using mean weight, which displayed similar trends but are slightly more spread out (Fig 4 in S1 Appendix). This difference can be attributed to the spatio-temporal model predicting biomass in years and locations with sampling gaps.

### Fishing effect

Our findings suggest a negative relationship between snow crab biomass and fishing (measured as catch) in divisions 3L and 3N, although these relationships were not statistically significant (*p* = 0.1 *and* 0.9, respectively). Similarly, we observed a negative relationship between yellowtail flounder biomass and fishing in divisions 3N and 3O. These relationships were also not statistically significant, although the fishing effect was more pronounced in division 3N (*p* = 0.08 *and* 0.48, respectively) (Fig 5 in S1 Appendix).

## Discussion

Our findings highlight the importance of considering spatial heterogeneity in fisheries survey data, as the spatial component accounted for the majority of observed variance for all three species (see Table 2). The spatial component explains variance that is not accounted for by the covariates depth and temperature. We observed that spatial effects differed between the occurrence and biomass processes for snow crab and Atlantic cod, but not for yellowtail flounder. These differences have been found in other studies and may be indicating spatial differences between species life stages, which are ignored in the occurrence analysis but get weighted in the biomass analysis [43]. Likewise, we noted a disparity in the relationship between biomass and depth for snow crab in both the occurrence and biomass processes, which is likely linked to the preference of adult snow crab for deeper waters [44]. Biomass hotspots are more restrictive than occurrence hotspots since biomass is higher only in areas with suitable conditions (e.g., environmental, reduce competition, prey availability), whereas individuals have a wide spatial range where they can be found [43].

The new biomass indices presented in this study overcome issues related with gaps in sampling by interpolating among missing data points. Missing data were important in the years 2015 and 2017 due to incomplete sampling during those periods. [21, 22]. They also account for species habitat-presence (i.e., depth and temperature) when predicting in unsampled locations and address spatio-temporal correlation. Predicted model biomass estimates align closely with those obtained using strata-based methods, with some disagreements for snow crab and Atlantic cod. In the case of snow crab, the assessment of the stock is done using data from the fall trawl survey. This is because spring data are considered to be less reliable because certain population components may be sampled relatively poorly during this time, coinciding with the mating and moulting periods [45]. In our analysis, we applied the conversion factor used to account for low catchability of snow crab in the fall bottom trawl survey [39, 40]. The development of this conversion factor was created using data from 2000 to 2016. Thus, the disparities observed in the biomass indices, particularly during the early stages of the temporal series, could potentially be explained by the exclusion of certain years and differences between fall and spring data. Spring data correspond to the pre-fishery season; the higher biomass observed in the new biomass index compared to the fall index could also be attributed to this. For Atlantic cod, differences are mostly due to the comparison of 3NO to 3LNO Atlantic cod.

The models used in the assessment of all stocks included in our analysis, except 3NO Atlantic cod, require biomass indices as input (snow crab assessment uses biomass trends, yellowtail flounder a Schaefer surplus production model and 2J3KL Atlantic cod a state-space model). Therefore, the new biomass indices developed in the present study could be used in the assessment of the species. Use of spatio-temporal indices to fit stock assessments has been shown to improve estimate precision compared to design-based indices [12, 46]. Consequently, this approach has been adopted by governmental bodies such as DFO and the United States National Oceanic and Atmospheric Administration (NOAA) to conduct the assessment of species such as northern shrimp (*Pandalus borealis*) [47] and yelloweye rockfish (*Sebastes ruberrimus*) [48]. However, it is important to acknowledge that calculating these indices is computationally intensive and presents implementation challenges. For example, spatial confounding (i.e., unaccounted spatial effects influencing the relationships between predictors and response variables) may exist, leading to bias in predictions [49]. Thus, accurately defining the spatial component is crucial in spatio-temporal models [50].

Centre of gravity has been used to evaluate the impacts of climate, fishing pressure and other anthropogenic factors on the average location of marine populations [51, 52]. A shift in species centre of gravity may create challenges and risks for managing resources when species move outside of historical fishing areas or management boundaries [53, 54]. The temperature distribution over The Grand Banks is not uniform, with the south and the north warmer than the centre. Additionally, this area undergoes natural cyclical periods of cold and warmth [27]. Changes in species distribution (here reflected as changes in the centre of gravity) can be an early sign of water warming on The Grand Banks due to natural variability and/or to climate change [55, 56]. However, shifts in species distribution may also be influenced by other factors such as competition, prey availability, habitat degradation and fishing [51, 57]. Our results indicate that snow crab is the most sensitive species to warming as its biomass declined as temperature increases, in agreement with other observations in Newfoundland and Labrador, where cold events have been associated with higher recruitment of snow crab [58, 59]. Yellowtail flounder tolerates a wide range of temperatures, with a preference for 3 ºC. This agrees with the literature, stating that yellowtail flounder can survive wide fluctuations in temperature [60, 61]. The persistence in the yellowtail centre of gravity on The Grand Banks is likely related to a weak current regime allowing for the retention of eggs and larvae in the southern part of The Grand Banks [62]—hypothesized as a nursery ground [63]. Atlantic cod has preference for warmer waters compared to yellowtail flounder and snow crab, favoring temperatures around 5 ºC. The northward shift in the center of gravity of Atlantic cod could be explained by a faster recovery of the 3L component of the 2J3KL stock, whereas the 3NO component may been experiencing a slower recovery. However, it may also be related to a northern shift of the species as a response to warming in the region due to a northern shift of the Gulf Stream [55].

Management of natural resources is a complex task that should consider ecological processes and how they relate to administrative boundaries. When management units are solely defined based on these administrative boundaries, decisions can have unintended consequences for the ecosystem [64]. Our analysis of Atlantic cod showed a continuous distribution over The Grand Banks during years of higher biomass (Fig 6 in S1 Appendix), indicating hyperaggregation behavior (i.e., aggregation of fish in a location during a period of low abundance [65]). While this behavior can decrease individual competition and maximize fitness, it can also increase the vulnerability of the species to fishing because of range contraction [66]. Previous studies have documented the mixing of Atlantic cod stocks [67, 68], suggesting that a joint assessment of the population and management decisions at the 2J3KL and 3NO management units, similar to the approach taken with snow crab, would be prudent. However, the pursuit of such collaborative efforts may face institutional barriers, given that these two Atlantic cod stocks fall under the purview of distinct regulatory bodies, namely DFO and NAFO. NAFO is a regional fisheries management organization responsible of the management of high seas fishery resources while DFO manages resources within the Canadian economic exclusive zone. Both entities apply the precautionary approach and reference points to manage the resources [69, 70], however, differences in management strategies, regulatory frameworks, and governance structures across these governing entities may exist.

Ocean warming (natural or driven by climate change) has been identified as the primary driver of snow crab decline in Newfoundland, while fishing and competition may have had localized impacts [71]. Overfishing is generally blamed for the decline of yellowtail flounder stocks in the early 1990s, but the productivity of the species was also strongly influenced by climatic conditions during the collapse and subsequent recovery [63]. In the present analysis, even though we could not directly account for the fishing effect in our models, we assessed the effect of fishing on species biomass at a coarser spatial resolution—the NAFO division level. We found that, even though the negative effect of fishing (i.e., landed catches) on species biomass was important in certain divisions (such as 3L and 3N for snow crab, and 3N for yellowtail flounder), these relationships were not statistically significant (refer to Fig 5 in S1 Appendix). The divisions with strongest fishing effect overlaped with those in which species had a biomass hotspot. This is not surprising, since fishers harvest on aggregations of individuals and not homogeneously across the entire area. However, the non-significance (*p* > 0.05) of the fishing effect on the new biomass indices suggests that additional factors are likely contributing to the observed decline in species biomass.

Our analysis is based on spring survey data, and therefore, the distribution patterns we observed may differ during other seasons. Species distribution is highly influenced by seasonal cycles, particularly in temperate areas, due to variations in environmental factors, light availability, and nutrient supply [72, 73]. Atlantic cod of the 2J3KL stock seek refuge near the continental shelf edge in winter and move to shallow coastal waters and onto The Grand Banks plateau during spring and summer [74]. Similarly, snow crab undergo seasonal migrations related to moulting and mating [44]. It is likely that we are missing information on species affinity for habitats that rely on seasonal variations. In addition, we used aggregated size and sex information of species to predict biomass. Distribution differences among species life stages may exist, including potential shifts in habitat preferences between juvenile and adult stages [75].

As species ranges shift in different directions and rates, it is likely that predator-prey interactions will also change [76]. The availability and abundance of prey directly impacts the population dynamics and distribution of predators [77, 78]. When prey species become scarce, predators may experience declines in body condition and overall population size [79]. The diminishing abundance of capelin, the primary prey of Atlantic cod, has been identified as a contributing factor to the decline in Atlantic cod growth potential [79] and body condition [80] of the 2J3KL stock. Fisheries harvest of other prey items of the Atlantic cod (i.e. snow crab and northern shrimp) could further exacerbate the issue of food limitation, hampering stock recovery [79, 80]. On the other hand, predators like Atlantic cod can play a regulatory role in shaping prey populations. In the Barents Sea, Atlantic cod has been identified as a regulator of the snow crab population, impacting both its distribution and productivity [81, 82]. A comparable phenomenon could potentially exist between these species on The Grand Banks. The examination of the distribution maps (Fig 2g and 2u) reveals limited overlap between Atlantic cod and snow crab in The Grand Banks. The limited overlap could be primarily attributed to the preference of snow crab for lower temperatures (below 0 ºC), however snow crab distribution may also be regulated by Atlantic cod presence. Future studies could explore predator-prey dynamics and species competition using alternative methodologies such as joint species distribution models [83, 84]. Additionally, incorporating substrate type in future research could provide valuable insights into the habitat preferences of demersal and benthic species of The Grand Banks. Future research could enhance ecosystem understanding by incorporating predation-prey interactions [85, 86], integrate multiple sources of data [13] and forecast climate change impacts on The Grand Banks under different emissions scenarios [87]. Our work could serve as a foundation for the development of spatial management strategies, including the establishment of conservation areas and spatial closures [88, 89].

## Conclusions

The introduction of novel indices offers a practical avenue for informed decision-making and underscores the importance of comprehensive approaches to fisheries management.

Our study revealed a continuous distribution of Atlantic cod across The Grand Banks and a possible northern shift of the species, emphasizing the need for joint management of the 2J3KL and 3NO stocks. The research presented here holds promise for enhancing the sustainability of Canadian fisheries by improving our understanding of the interactions between environmental variables and species distributions.

## Supporting information

S1 Appendix (DOCX)

S2 Appendix Model outputs and validation. (DOCX)
