# Supplementary material for: Decadal changes in biomass and distribution of key fisheries species on Newfoundland’s Grand Banks
Source: PLoS One. 2024 Apr 1;19(4):e0300311. doi: 10.1371/journal.pone.0300311 (PMC10984460; doi:10.1371/journal.pone.0300311)
Supplement: S1 Appendix — (DOCX) [file pone.0300311.s001.docx]

**Appendix S1**

**Decadal changes in biomass and distribution of key fisheries species on Newfoundland’s Grand Banks.**

Raquel Ruiz-Diaz^1^, Maria Grazia Pennino^2^, Jonathan Fisher^1^, Tyler Eddy^1^


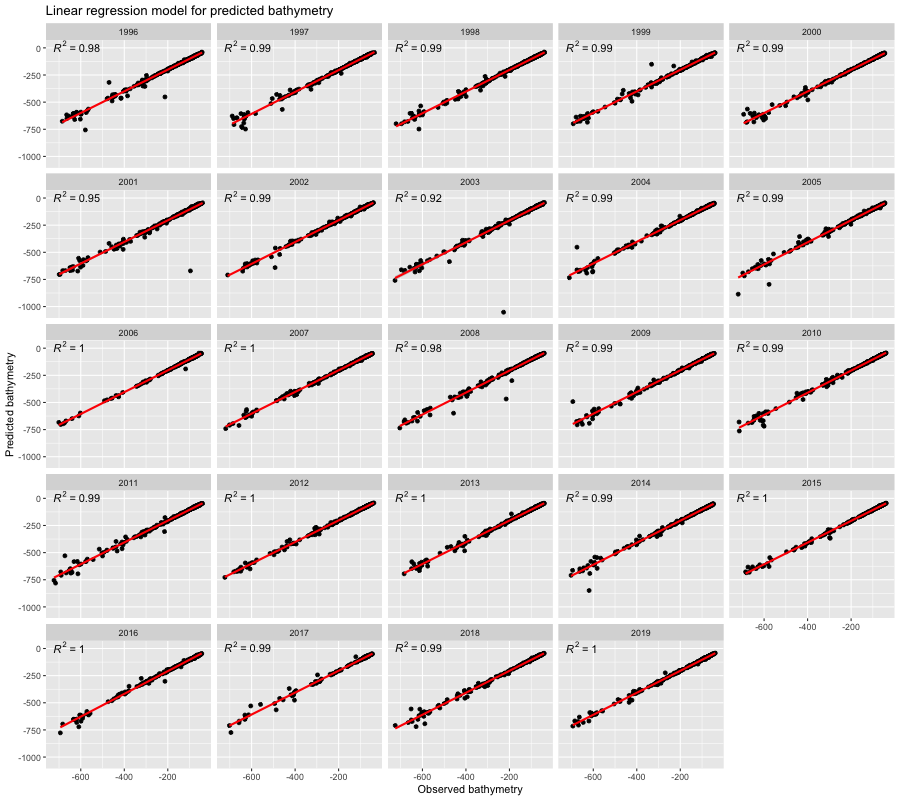
**Correlation between covariates observation and data use to predict.**

Figure 1. Correlation between observed bathymetry (obtained from the bottom trawl survey) and GEBCO bathymetry used to predict.


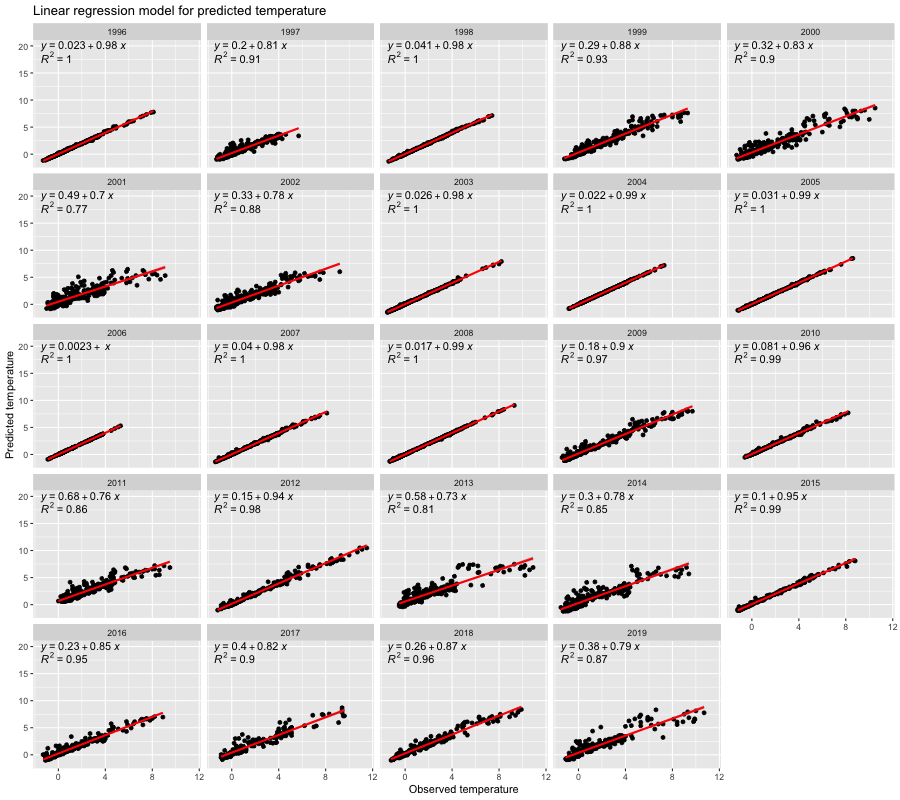


Figure 2. Correlation between observed bottom temperature (obtained from the bottom trawl survey) and DFO interpolated bottom temperature data used to predict.

**Delaunay triangulation mesh**


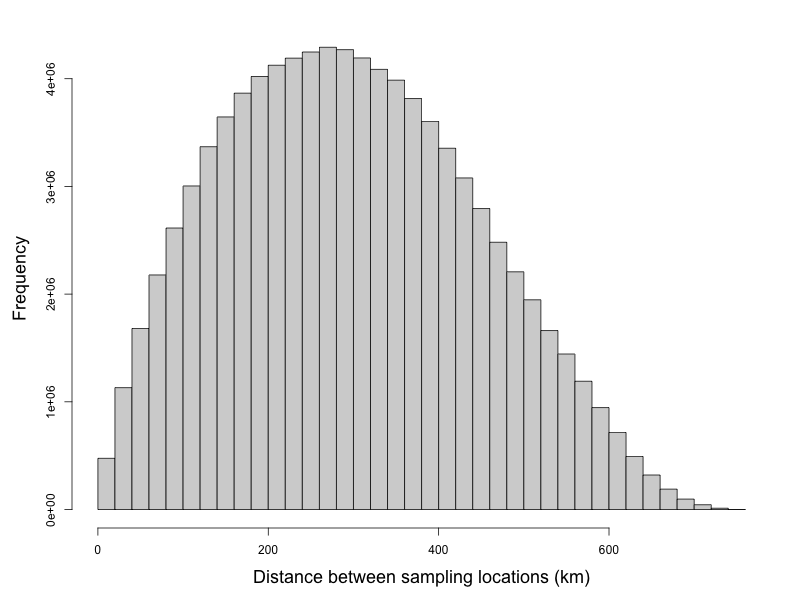
**
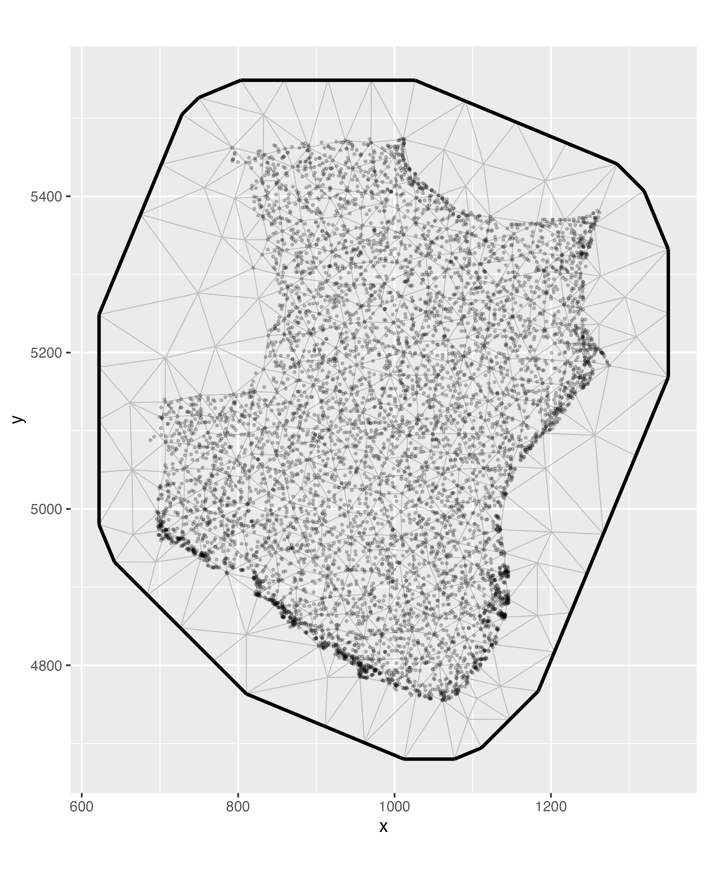
**

Figure 3. Left graph displays the Delaunay triangulation mesh used in the models with a cutoff = 20 km. Grey dots indicate observations. Right graphs shows an histogram of the distance between sampling sites in Km.

**Mesh description**

fm_mesh_2d object:

Manifold: R2

V / E / T: 467 / 1359 / 893

Euler char.: 1

Constraints: 39 boundary edges (1 group: 0), 0 boundary edges

Bounding box: ( 622.1104,1350.0463) x (4679.981,5548.451) x (0,0)

Basis d.o.f.: 467

**Changes in the centre of gravity calculated from the dataset.**

**
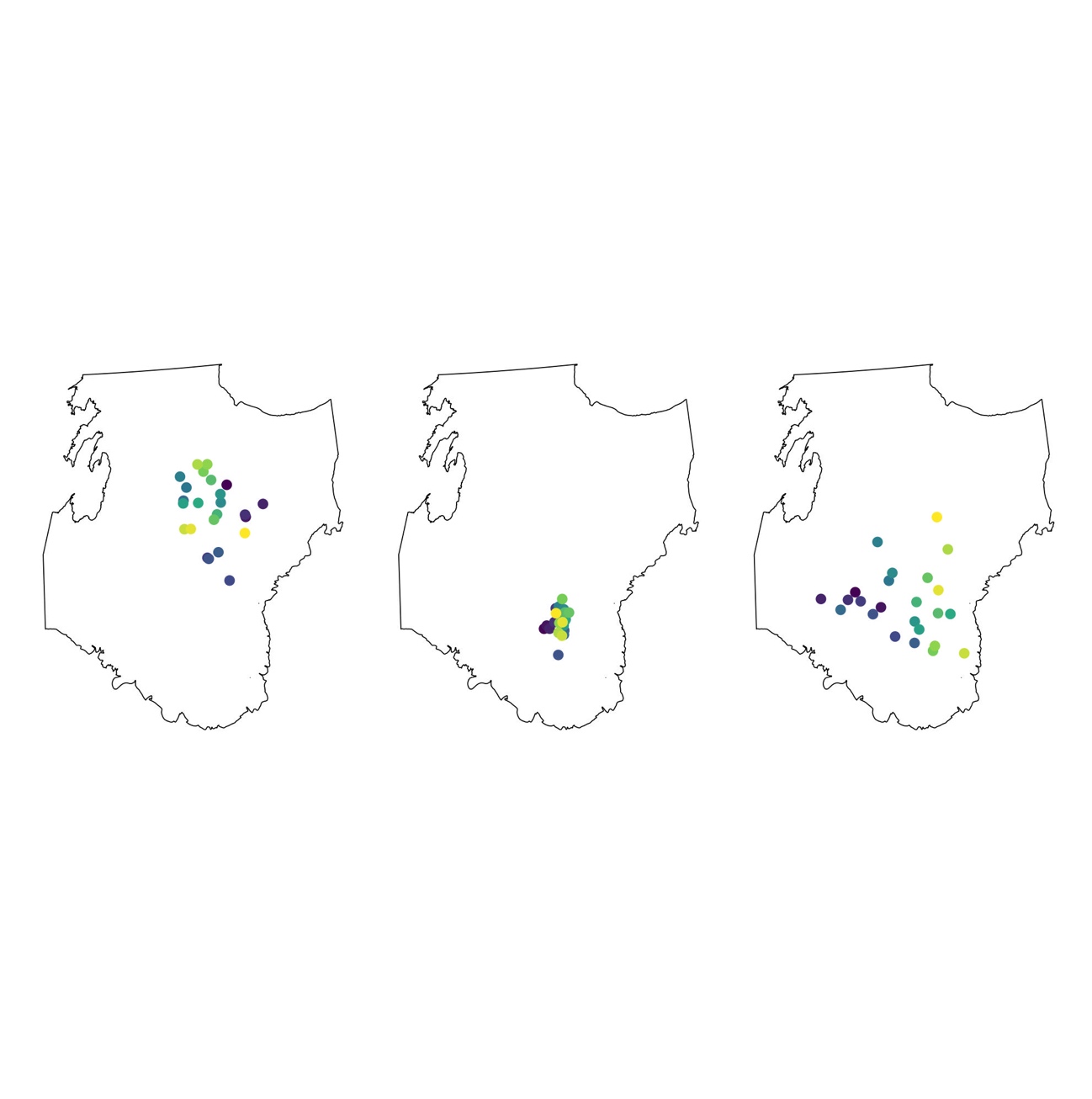
**

Figure 4. Changes in the centre of gravity of snow crab (left), yellowtail flounder (center) and Atlantic cod (right)

**Relationship between species biomass and fishing effort at division level.**

Figure 5. linear relationship between biomass and catches of snow crab (a) and yellowtail flounder (b) calculated at division level (3L, 3N and 3O)

**Delta_gamma biomass prediction per year**

*Atlantic cod predicted biomass*

Figure 6. Maps of Atlantic cod biomass distribution over the Grand Banks obtained from the combined prediction of the delta_gamma model. Biomass is in log scale.

*Snow crab predicted biomass*

Figure 7. Maps of snow crab biomass distribution over the Grand Banks obtained from the combined prediction of the delta_gamma model. Biomass is in log scale.

*Yellowtail flounder predicted biomass*

Figure 8. Maps of yellowtail flounder biomass distribution over the Grand Banks obtained from the combined prediction of the delta_gamma model. Biomass is in log scale.
