# Supplementary material for: Decadal changes in biomass and distribution of key fisheries species on Newfoundland’s Grand Banks
Source: PLoS One. 2024 Apr 1;19(4):e0300311. doi: 10.1371/journal.pone.0300311 (PMC10984460; doi:10.1371/journal.pone.0300311)
Supplement: S2 Appendix — (DOCX) [file pone.0300311.s002.docx]

**Appendix S2**

**Decadal changes in biomass and distribution of key fisheries species on Newfoundland’s Grand Banks.**

Raquel Ruiz-Diaz^1^, Maria Grazia Pennino^2^, Jonathan Fisher^1^, Tyler Eddy^1^

# Model outputs and validation

## Snow crab model outputs

Spatiotemporal model fit by ML ['sdmTMB']

Formula: biomass ~ 0 + as.factor(year) + s(depth_log) + s(tempatfishing)

Mesh: mesh

Time column: year

Data: data

Family: delta_gamma(link1 = 'logit', link2 = 'log')

Delta/hurdle model 1: -----------------------------------

Family: binomial(link = 'logit')

coef.est coef.se

as.factor(year)1996 0.84 0.65

as.factor(year)1997 0.96 0.65

as.factor(year)1998 1.15 0.64

as.factor(year)1999 2.09 0.65

as.factor(year)2000 1.83 0.65

as.factor(year)2001 1.87 0.65

as.factor(year)2002 2.78 0.66

as.factor(year)2003 1.87 0.65

as.factor(year)2004 1.04 0.64

as.factor(year)2005 0.65 0.64

as.factor(year)2006 -0.34 0.66

as.factor(year)2007 -0.68 0.64

as.factor(year)2008 -0.56 0.64

as.factor(year)2009 0.45 0.64

as.factor(year)2010 0.56 0.64

as.factor(year)2011 0.75 0.65

as.factor(year)2012 0.07 0.65

as.factor(year)2013 -0.04 0.64

as.factor(year)2014 0.02 0.65

as.factor(year)2015 -0.50 0.67

as.factor(year)2016 -0.53 0.65

as.factor(year)2017 -0.38 0.67

as.factor(year)2018 -0.51 0.65

as.factor(year)2019 -0.22 0.64

sdepth_log 5.75 6.84

stempatfishing -4.48 0.79

Smooth terms:

Std. Dev.

sds(depth_log) 5

sds(tempatfishing) 0

Spatiotemporal AR1 correlation (rho): 0.86

Matern range: 145.52

Spatial SD: 1.82

Spatiotemporal SD: 1.22

Delta/hurdle model 2: -----------------------------------

Family: Gamma(link = 'log')

coef.est coef.se

as.factor(year)1996 4.38 0.33

as.factor(year)1997 4.02 0.33

as.factor(year)1998 4.06 0.33

as.factor(year)1999 3.83 0.33

as.factor(year)2000 3.31 0.33

as.factor(year)2001 3.28 0.33

as.factor(year)2002 3.29 0.33

as.factor(year)2003 3.43 0.33

as.factor(year)2004 2.61 0.33

as.factor(year)2005 2.66 0.34

as.factor(year)2006 2.36 0.36

as.factor(year)2007 2.66 0.35

as.factor(year)2008 2.55 0.35

as.factor(year)2009 3.08 0.34

as.factor(year)2010 2.49 0.34

as.factor(year)2011 2.83 0.34

as.factor(year)2012 2.64 0.35

as.factor(year)2013 2.33 0.34

as.factor(year)2014 2.38 0.35

as.factor(year)2015 1.62 0.37

as.factor(year)2016 1.17 0.35

as.factor(year)2017 1.76 0.40

as.factor(year)2018 1.67 0.36

as.factor(year)2019 2.06 0.35

sdepth_log 8.43 4.60

stempatfishing 2.84 4.94

Smooth terms:

Std. Dev.

sds(depth_log) 3.28

sds(tempatfishing) 1.57

Dispersion parameter: 0.93

Spatiotemporal AR1 correlation (rho): 0.71

Matern range: 97.00

Spatial SD: 1.15

Spatiotemporal SD: 1.22

ML criterion at convergence: 8017.538

## Snow crab model diagnostic plots

Figure 1. Histogram (left) and quantile-quantile normality (QQ) plot for snow crab biomass model (right) with random effects estimated with MCMC while fixing fixed effects at their MLE values.


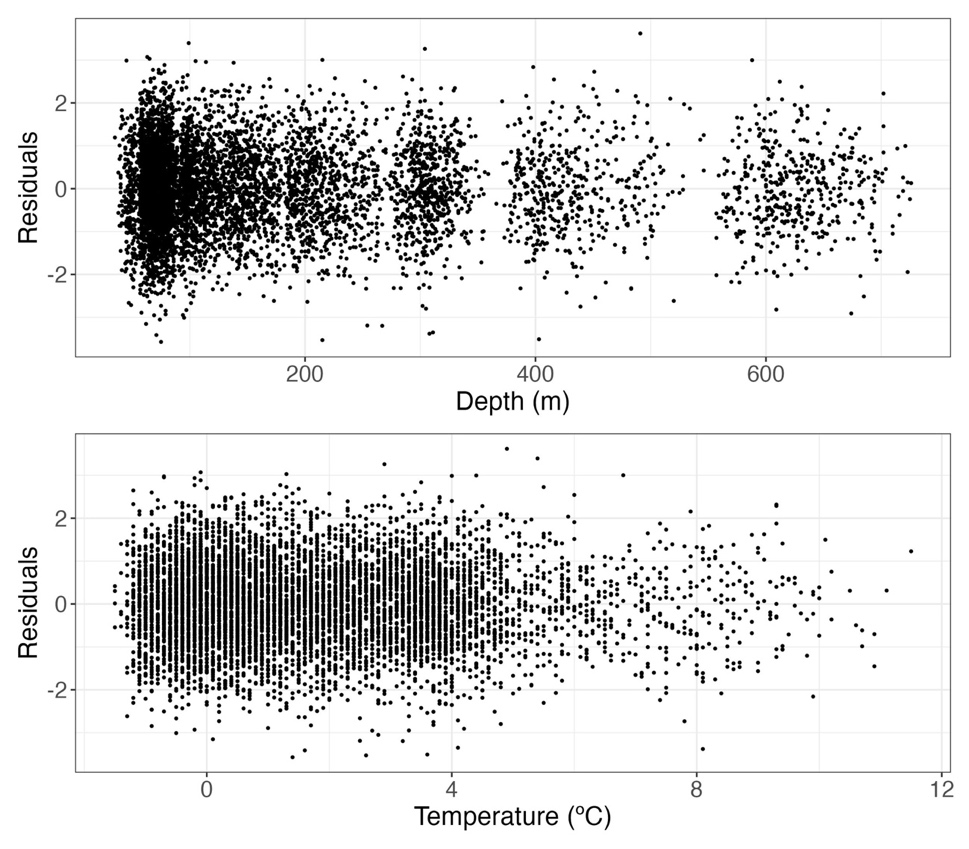


Figure 2. Plot of snow crab biomass model residuals against each explanatory variable, depth (upper plot) and temperature (lower plot)

Figure 3. Spatial and temporal distribution of residuals based on snow crab biomass model.

## Yellowtail flounder model output

Spatiotemporal model fit by ML ['sdmTMB']

Formula: biomass ~ 0 + as.factor(year) + s(depth_log) + s(tempatfishing)

Mesh: mesh

Time column: year

Data: data

Family: delta_gamma(link1 = 'logit', link2 = 'log')

Delta/hurdle model 1: -----------------------------------

Family: binomial(link = 'logit')

coef.est coef.se

as.factor(year)1996 -4.85 1.47

as.factor(year)1997 -4.75 1.51

as.factor(year)1998 -6.53 1.47

as.factor(year)1999 -3.97 1.45

as.factor(year)2000 -3.45 1.45

as.factor(year)2001 -4.78 1.46

as.factor(year)2002 -5.44 1.46

as.factor(year)2003 -4.36 1.46

as.factor(year)2004 -4.61 1.44

as.factor(year)2005 -3.82 1.43

as.factor(year)2006 -0.65 1.44

as.factor(year)2007 -2.37 1.42

as.factor(year)2008 -1.88 1.41

as.factor(year)2009 -4.88 1.44

as.factor(year)2010 -3.33 1.42

as.factor(year)2011 -2.11 1.41

as.factor(year)2012 -1.11 1.41

as.factor(year)2013 -1.95 1.41

as.factor(year)2014 -1.87 1.42

as.factor(year)2015 -3.51 1.45

as.factor(year)2016 -4.05 1.43

as.factor(year)2017 -3.52 1.48

as.factor(year)2018 -3.05 1.43

as.factor(year)2019 -3.62 1.44

sdepth_log -13.32 6.31

stempatfishing -2.56 3.62

Smooth terms:

Std. Dev.

sds(depth_log) 2.85

sds(tempatfishing) 1.61

Spatiotemporal AR1 correlation (rho): 0.82

Matern range: 231.86

Spatial SD: 2.69

Spatiotemporal SD: 1.55

Delta/hurdle model 2: -----------------------------------

Family: Gamma(link = 'log')

coef.est coef.se

as.factor(year)1996 3.22 0.54

as.factor(year)1997 3.26 0.55

as.factor(year)1998 3.20 0.54

as.factor(year)1999 4.42 0.52

as.factor(year)2000 4.25 0.52

as.factor(year)2001 3.77 0.54

as.factor(year)2002 2.90 0.54

as.factor(year)2003 3.89 0.53

as.factor(year)2004 3.74 0.52

as.factor(year)2005 4.56 0.51

as.factor(year)2006 5.09 0.51

as.factor(year)2007 4.70 0.50

as.factor(year)2008 4.81 0.50

as.factor(year)2009 4.00 0.52

as.factor(year)2010 4.56 0.51

as.factor(year)2011 4.83 0.50

as.factor(year)2012 5.31 0.50

as.factor(year)2013 4.73 0.50

as.factor(year)2014 4.43 0.51

as.factor(year)2015 4.28 0.52

as.factor(year)2016 3.36 0.51

as.factor(year)2017 3.48 0.53

as.factor(year)2018 4.35 0.51

as.factor(year)2019 3.79 0.52

sdepth_log 0.58 4.92

stempatfishing -0.78 1.94

Smooth terms:

Std. Dev.

sds(depth_log) 2.42

sds(tempatfishing) 0.78

Dispersion parameter: 1.25

Spatiotemporal AR1 correlation (rho): 0.73

Matern range: 128.11

Spatial SD: 1.25

Spatiotemporal SD: 1.55

ML criterion at convergence: 12570.374

## Yellowtail flounder diagnostic plots

Figure 4. Histogram (left) and quantile-quantile normality (QQ) plot for yellowtail flounder biomass model (right) with random effects estimated with MCMC while fixing fixed effects at their MLE values


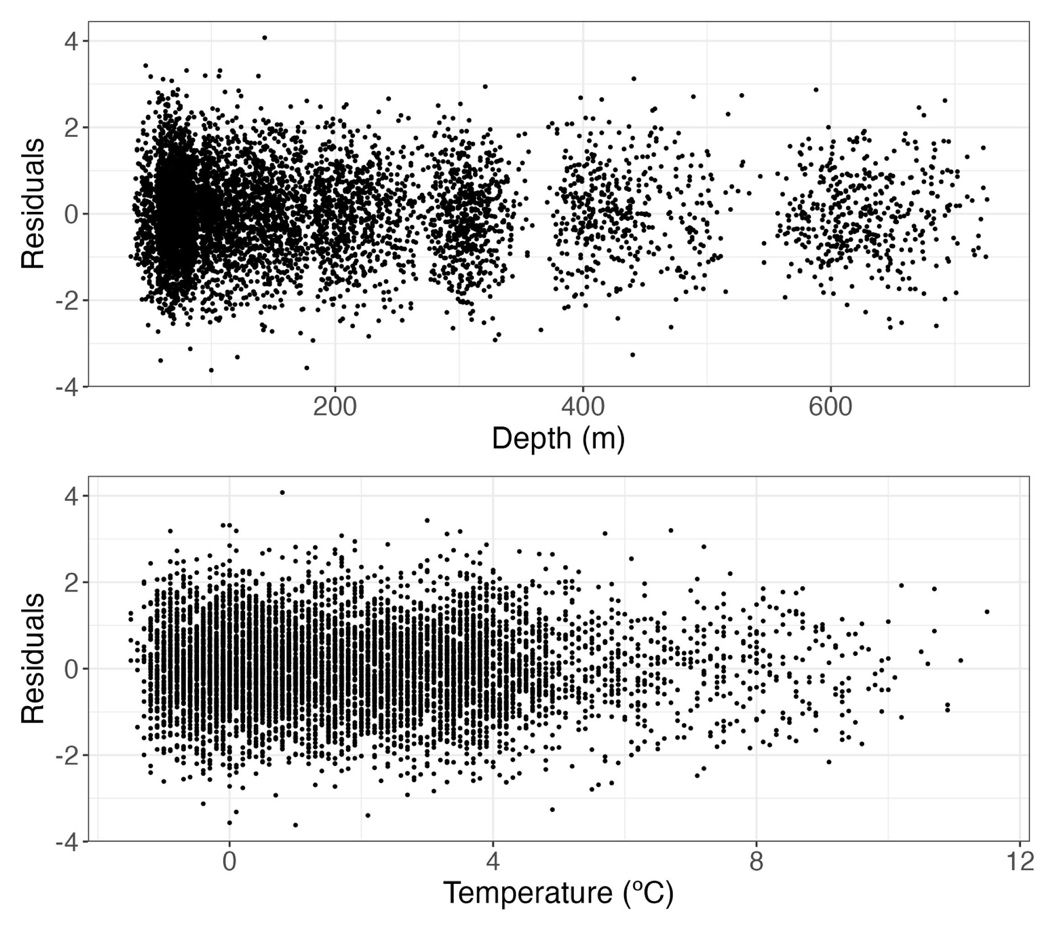


Figure 5. Plot of yellowtail flounder biomass model residuals against each explanatory variable, depth (upper plot) and temperature (lower plot)

Figure 6. Spatial and temporal distribution of residuals based on yellowtail flounder biomass model

## Atlantic cod model output

Spatiotemporal model fit by ML ['sdmTMB']

Formula: biomass ~ 0 + as.factor(year) + s(depth_log) + s(tempatfishing)

Mesh: mesh

Time column: year

Data: data

Family: delta_gamma(link1 = 'logit', link2 = 'log')

Delta/hurdle model 1: -----------------------------------

Family: binomial(link = 'logit')

coef.est coef.se

as.factor(year)1996 0.27 0.50

as.factor(year)1997 -0.35 0.51

as.factor(year)1998 -0.41 0.50

as.factor(year)1999 0.85 0.51

as.factor(year)2000 0.73 0.51

as.factor(year)2001 0.93 0.51

as.factor(year)2002 0.14 0.51

as.factor(year)2003 0.10 0.51

as.factor(year)2004 -0.55 0.51

as.factor(year)2005 0.52 0.51

as.factor(year)2006 1.31 0.54

as.factor(year)2007 1.14 0.52

as.factor(year)2008 1.07 0.51

as.factor(year)2009 0.53 0.51

as.factor(year)2010 0.38 0.51

as.factor(year)2011 0.13 0.51

as.factor(year)2012 1.20 0.52

as.factor(year)2013 1.37 0.52

as.factor(year)2014 1.24 0.52

as.factor(year)2015 1.12 0.53

as.factor(year)2016 1.19 0.51

as.factor(year)2017 -0.18 0.56

as.factor(year)2018 0.66 0.51

as.factor(year)2019 0.82 0.51

sdepth_log -7.72 7.90

stempatfishing 2.21 4.73

Smooth terms:

Std. Dev.

sds(depth_log) 8.94

sds(tempatfishing) 3.04

Matern range: 189.74

Spatial SD: 1.04

Spatiotemporal SD: 0.85

Delta/hurdle model 2: -----------------------------------

Family: Gamma(link = 'log')

coef.est coef.se

as.factor(year)1996 4.01 0.33

as.factor(year)1997 4.37 0.35

as.factor(year)1998 4.51 0.34

as.factor(year)1999 4.71 0.33

as.factor(year)2000 4.57 0.33

as.factor(year)2001 4.49 0.33

as.factor(year)2002 3.89 0.34

as.factor(year)2003 4.22 0.34

as.factor(year)2004 4.00 0.34

as.factor(year)2005 4.73 0.33

as.factor(year)2006 5.07 0.35

as.factor(year)2007 4.83 0.33

as.factor(year)2008 4.97 0.33

as.factor(year)2009 4.61 0.34

as.factor(year)2010 4.31 0.33

as.factor(year)2011 4.46 0.33

as.factor(year)2012 5.33 0.33

as.factor(year)2013 5.55 0.33

as.factor(year)2014 5.60 0.34

as.factor(year)2015 5.03 0.35

as.factor(year)2016 4.12 0.33

as.factor(year)2017 3.71 0.41

as.factor(year)2018 4.60 0.34

as.factor(year)2019 4.19 0.33

sdepth_log -5.21 4.98

stempatfishing 1.32 3.20

Smooth terms:

Std. Dev.

sds(depth_log) 3.07

sds(tempatfishing) 1.68

Dispersion parameter: 0.75

Matern range: 95.97

Spatial SD: 0.98

Spatiotemporal SD: 0.85

ML criterion at convergence: 13000.057

## Atlantic cod diagnostic plots

Figure 7. Histogram (left) and quantile-quantile normality (QQ) plot for Atlantic cod biomass model (right) with random effects estimated with MCMC while fixing fixed effects at their MLE values


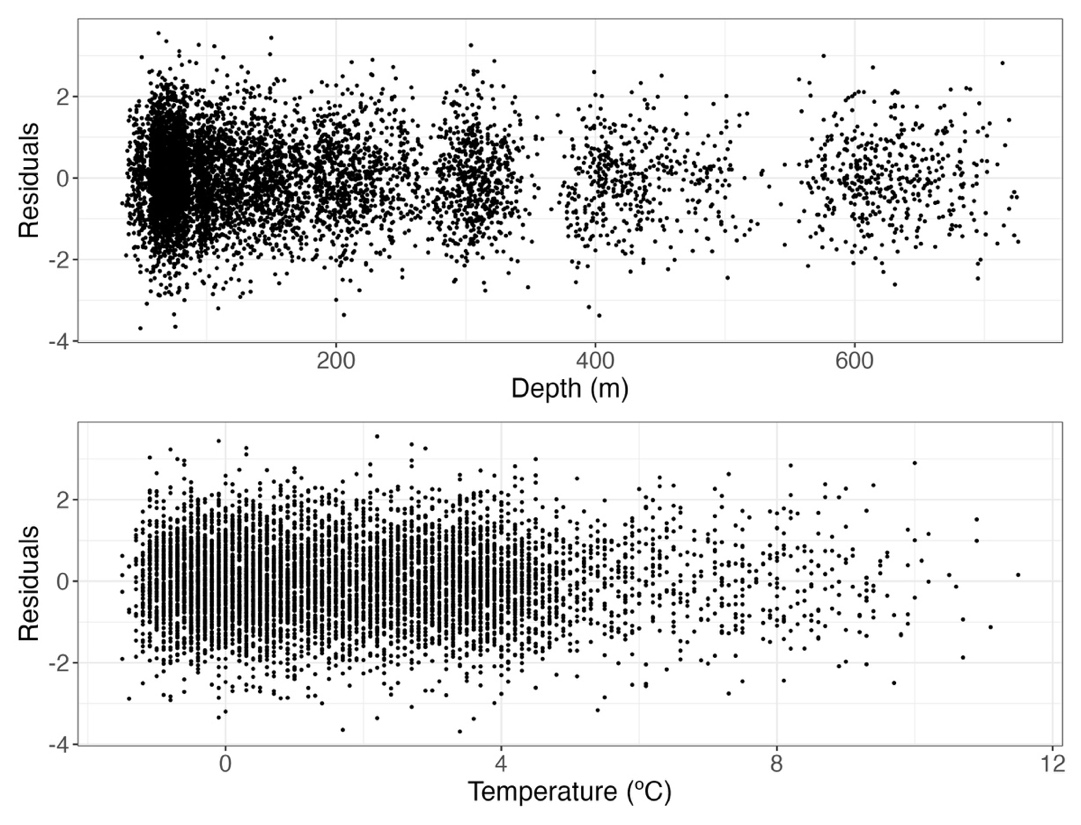


Figure 8. Plot of Atlantic cod biomass model residuals against each explanatory variable, depth (upper plot) and temperature (lower plot)

Figure 9. Spatial and temporal distribution of residuals based on Atlantic cod biomass model
